# Magnetic Charge Fingerprints in the Spin-Wave Spectrum of Three-Dimensional Artificial Spin Ice

**DOI:** 10.1021/acs.nanolett.6c00670

**Published:** 2026-05-04

**Authors:** Chandan Kumar, Amrit Kumar Mondal, Sreya Pal, Sayan Mathur, Jay R. Scott, Arjen van den Berg, Adekunle O. Adeyeye, Sam Ladak, Anjan Barman

**Affiliations:** † Department of Condensed Matter and Materials Physics, 30178 S. N. Bose National Centre for Basic Sciences, Block JD, Sector III, Salt Lake, Kolkata 700106, India; ‡ Technical Research Centre, S. N. Bose National Centre for Basic Sciences, Block JD, Sector III, Salt Lake, Kolkata 700106, India; § Department of Physics, 3057 Durham University, Durham DH1 3LE, United Kingdom; ∥ School of Physics and Astronomy, 2112 Cardiff University, Cardiff CF24 3AA, United Kingdom; ⊥ Department of Physics, School of Natural Sciences, Shiv Nadar Institution of Eminence (Delhi NCR), Dadri, Uttar Pradesh 201314, India

**Keywords:** 3D artificial spin ice, Magnetic charge states, Spin-wave spectroscopy, Brillouin light scattering, Reconfigurable magnonics, 3D nanomagnetic networks

## Abstract

Three-dimensional artificial spin ice (3D-ASI) is a programmable nanoscale magnetic network showing emergent magnetic charge states with potential for reconfigurable dynamics. Here, we show that magnetic charge states in a 3D-ASI leave experimentally measurable charge-sensitive spectral signatures in the spin-wave spectrum, enabling charge-state readout via magnonics. Using Brillouin light scattering spectroscopy supported by micromagnetic simulations, we demonstrate that charged and charge-neutral vertices produce qualitatively distinct spin-wave spectra in a purely three-dimensional lattice. Spatially resolved mode analysis reveals that magnetic charges selectively control spin-wave localization and quantization, establishing a direct link between vertex microstate and dynamic response. We further demonstrate that configurational anisotropy inherent to the 3D architecture allows spin-wave reconfiguration by varying the orientation of an applied field. These results position spin-wave spectroscopy as a dynamic, noninvasive probe of magnetic charge states and open pathways to reprogrammable, low-power 3D magnonic and neuromorphic devices.

## Graphical abstract

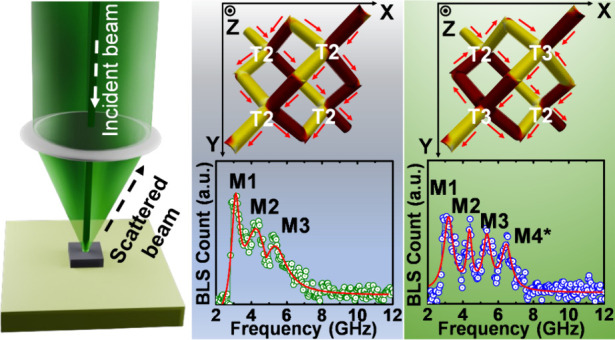

Artificial spin ice (ASI) systems are frustrated, engineered arrays of single-domain nanomagnets with magnetostatic interactions that dictate their ordering and ground state.^1,2^ The unique properties of these frustrated metamaterials have a wide range of applications in magnonics,^3,4^ reservoir computing,^5,6^ and data storage,^7^ and are also crucial for the fundamental understanding of captivating phenomena, including emergent magnetic monopoles,^8,9^ vertex-based frustration,^10,11^ phase transitions,^12^ and chiral dynamics.^13^ Previous exploration of ASI systems were mostly done in planar two-dimensional (2D) nanoarchitectures,^14^ thus transcending the limitations of 2D to explore the unique characteristics of these metamaterials in three-dimension (3D) is pivotal to explore the unique functionalities which is beyond the scope of 2D. Beyond traditional processes, advanced methods like two-photon lithography (TPL)^15^ and focused electron beam-induced deposition (FEBID)^16^ have revolutionized the fabrication of 3D nanostructures, enabling the study of complex systems such as the diamond bond lattice,^17^ double-helix,^18^ woodpile-structure,^19^ nanobridge^20^ and other complex 3D structures.^21^ In addition to conventional characterization techniques like magnetic force microscopy (MFM), advancements in nitrogen-vacancy magnetometry, X-ray and electron-based characterization techniques, along with improved computational analyses, have significantly progressed the fundamental understanding of 3D nanomagnets and 3D chiral spin textures.^22–26^ These developments have created fertile ground for exploring spin wave (SW) dynamics in these systems, driving the evolution of 3D magnonics.^27^

The investigation of SW dynamics in 3D-ASI structures is still at an early stage. Recent experimental studies on multilayered ASI nanoarrays^5,28^ and woodpile nanostructures^19,29^ have demonstrated promising modulation of SW characteristics. In parallel, micromagnetic simulation studies on buckyball^30^ and other 3D interconnected nanoarchitectures^31,32^ have revealed SW tuanability through the microstructure and the magnetization configuration. Recent advances in 3D visualization of SWs,^33^ coherent SW dynamics in nanoarchitectures,^34^ nanoscale SW confinements^35^ and magnetic charge propagation^36^ have further accelerated progress in the field. The development, fabrication, and characterization of 3D-ASI, along with emerging frontiers in 3D model systems and applications, have been comprehensively discussed in recent perspective article.^37^ Despite these advances, spectral fingerprinting of magnetically charged vertices has largely been confined to planar nanoarchitectures^5,38^ and simulated 3D-nanostructures.^30–32^ Experimental demonstrations of SW dynamics modulated by magnetic charge states in purely 3D-ASI systems remain largely absent from the literature. Moreover, experimental studies exploring magnetic-configuration-driven SW dynamics in 3D-ASI structures are still scarce. This research gap can be addressed by an experimental study on 3D-ASI structures with nanowires arranged into a diamond-bond 3D lattice geometry.^17^ Recent Monte Carlo study of this structure has revealed an exotic phase diagram consisting of the conventional ice-phase and monopole crystals consisting of single- and double-charged tiling. Magnetic imaging of experimental structures after in-plane demagnetization found crystallites of single magnetic charge superimposed upon an ice background.^39^ Furthermore, previous investigations of this system have demonstrated a range of rich phenomena, including magnetic charge propagation^40^ and the excitation of coherent SWs.^41^ This intriguing 3D system offers an exceptional opportunity to explore SW dynamics, which can be tuned by magnetic charged states and configurational anisotropy.

Geometrically frustrated 2D-ASI systems exhibit intricate SW dynamics due to the energy differences between various macrospin configurations, with these dynamics strongly dependent on the magnetic microstates.^38,42,43^ One of the intriguing features of ASI is its ability to tune the magnetic microstate through magnetization and demagnetization processes.^38,39^ Leveraging this property, we fabricated permalloy (Ni_81_Fe_19_) 3D-ASI samples using a combination of two-photon lithography and evaporation, which were subject to distinct magnetic field protocols. The sample fabrication is detailed in Section I of the Supporting Information. A scanning electron microscope image of a typical lattice with array dimensions of 50 × 50 × 10 μm^3^ is shown in Figure 1(a), with individual sublattices shown in Figure 1(b). Individual nanowires (length = 866 ± 10 nm) have a crescent shaped cross-section as shown schematically in Figure 1(c). A 3D perspective of a unit-cell is shown in Figure 1(d), allowing the depth of each sublattice to be identified. As shown in Figures 1(b) and 1(d), the lattice is composed of four vertically stacked sublattices (L1–L4). L1 forms the top layer, with nanowires aligned along an in-plane direction. Directly below it, L2 is oriented perpendicular to L1. L3 lies below L2 and is again parallel to L1, while the bottom layer, L4, is parallel to L2. This results in an alternating stack of orthogonal nanowire layers along the vertical direction. The magnetized sample was created by first applying and then reducing *H* along the surface termination sublattice (L1, Figure 1(b)). The demagnetized sample was obtained by spinning the 3D-ASI array in an oscillating *H* (details in Section I of the Supporting Information). Our previous work using MFM revealed distinct differences in vertex types: the magnetized sample^40,41^ contains only type-II (T2, charge-free) vertices, while the demagnetized sample exhibits magnetic charge crystallites superimposed upon an ice background. Overall, this yielded primarily coordination-number four vertices of type-III (T3) and T2 in an approximate ratio of 2:1, with few type-I (T1) and type-IV (T4) vertices.^39^ The samples studied here show qualitatively similar behavior as can be seen in the magnetization profiles for magnetized and demagnetized samples shown in Figure 1(e) and 1(f), respectively. The associated atomic force microscopy (AFM) and MFM can be found in the Supporting Information Figure S1.

**Figure 1:**
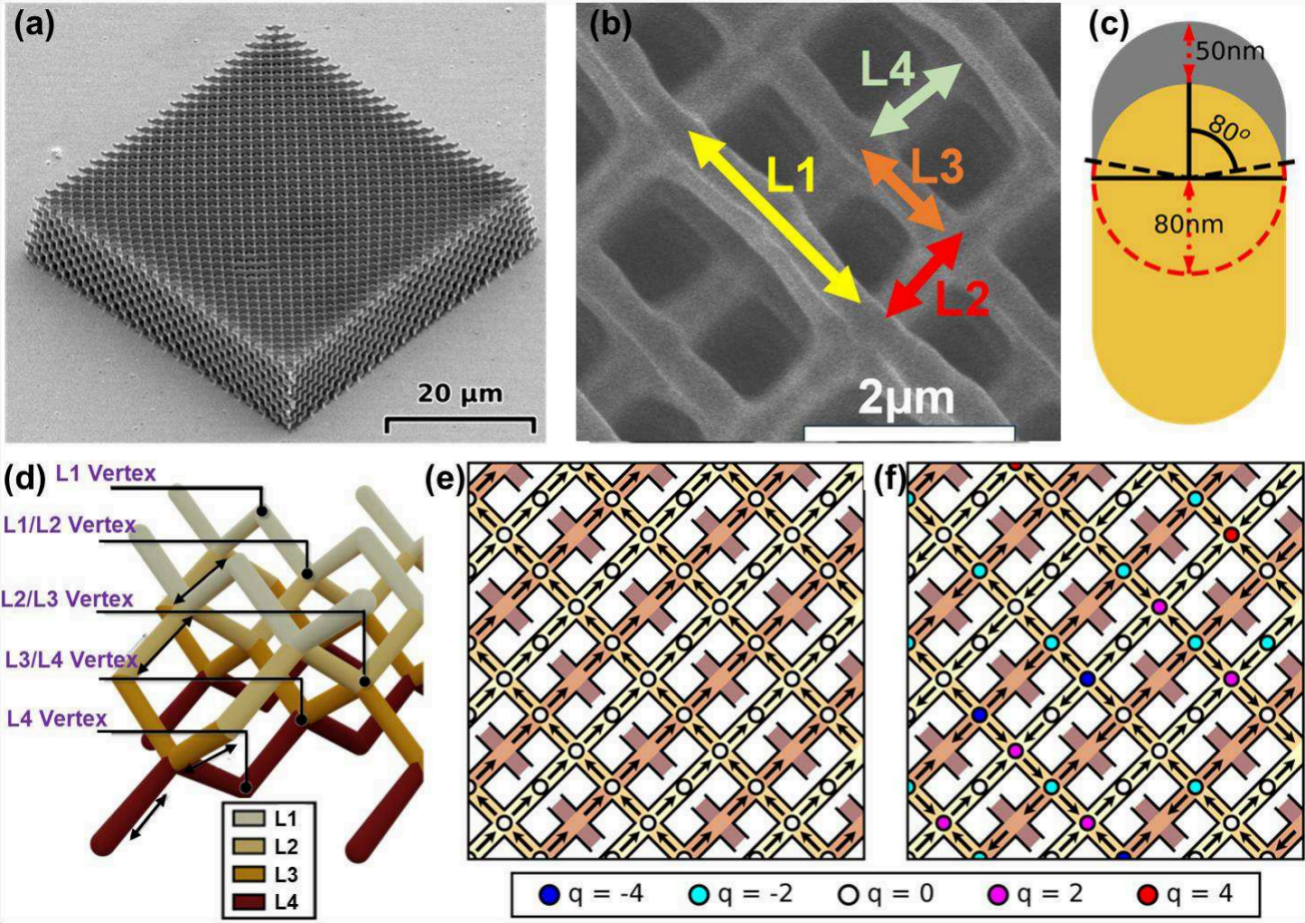
**(**a) Scanning electron microscopy image of a representative 3D-ASI sample, which takes the form of a diamond-bond lattice. (b) Zoomed image showing the individual sublattices. (c) Schematic of the nanowire cross-section. Magnetic material (gray) takes a crescent-shaped cross-section, upon the underlying polymer structure (yellow). (d) A unit cell of the 3D-ASI is shown schematically, with sublattice colored differently according to legend. (e) Arrow map showing the measured magnetization configuration of the magnetized sample, as based upon MFM measurements. Circles at the vertex indicate magnetic charge as described in the legend and individual sublattices are colored according to legend in (d). The sample has a two-in/two-out, T2 tiling at every vertex. (f) Arrow maps showing the measured magnetization configuration of the demagnetized sample, based upon MFM. The sample is found to possess charge-ordered regions, superimposed upon an ice T2 background.

The SW dynamics were probed using BLS spectroscopy with a ∼ 50 μm laser spot covering nearly the entire sample. As shown in Figure 1(b), all four sublattices are directly exposed to the incident beam, ensuring contributions from the complete 3D network. Although the permalloy forms a nanoscale coating on the polymer nanowire surface, it preserves the full 3D connectivity of the lattice. Consequently, the measured spectra represent a spatially averaged response of the entire 3D structure, rather than surface-confined excitations. The micromagnetic simulations were carried out using the GPU-accelerated micromagnetic simulation package MuMax3.^44^ Simulations were performed on the unit cell of the diamond-bond lattice of crescent-shaped nanowires with 2D periodic boundary conditions in the x–y plane, using charge distributions inspired by experimentally observed magnetized and demagnetized samples,^39–41^ as shown in Figures 2(a) and 2(b), respectively. The detailed description of the BLS spectroscopy and micromagnetic simulation methodology is provided in Section II of the Supporting Information. The modulation of SW excitation, driven by the distinct energy landscapes associated with charged and uncharged vertices,^39,40^ is clearly evident from the measured BLS spectra of the magnetized and demagnetized samples (Figures 2c and 2d, respectively). The magnetized sample exhibits three SW modes (M1, M2 and M3), while the demagnetized sample shows an additional mode (M4*), providing direct experimental evidence of magnetic charge-dependent SW excitation in a purely 3D nanomagnetic structure, previously reported only in simulations.^30–32^ The robustness of the additional mode is confirmed by consistent Gaussian and Lorentzian fits (see Supporting Information, Figure S3 and Table S1). The M4* mode reaches ∼77% of the M2/M3 intensity, providing a clear spectral signature of T3-containing environment in the demagnetized state. The observed differences between magnetized and demagnetized samples originate from the distinct demagnetizing-field landscapes of T2 and T3 vertices (see Supporting Information, Figure S4 and Table S2). Micromagnetic simulations reproduce these trends qualitatively (Figures 2(e) and 2(f)), with minor discrepancies in frequency and mode resolution arising from differences in sample morphology, measurement conditions, and excitation mechanisms typical of 3D systems.^19,41^ For example, the simulated splitting of the M1 mode is not experimentally resolved due to finite line width and ensemble averaging, while weaker high-frequency modes likely fall within the noise floor and remain below the detection limit.

**Figure 2:**
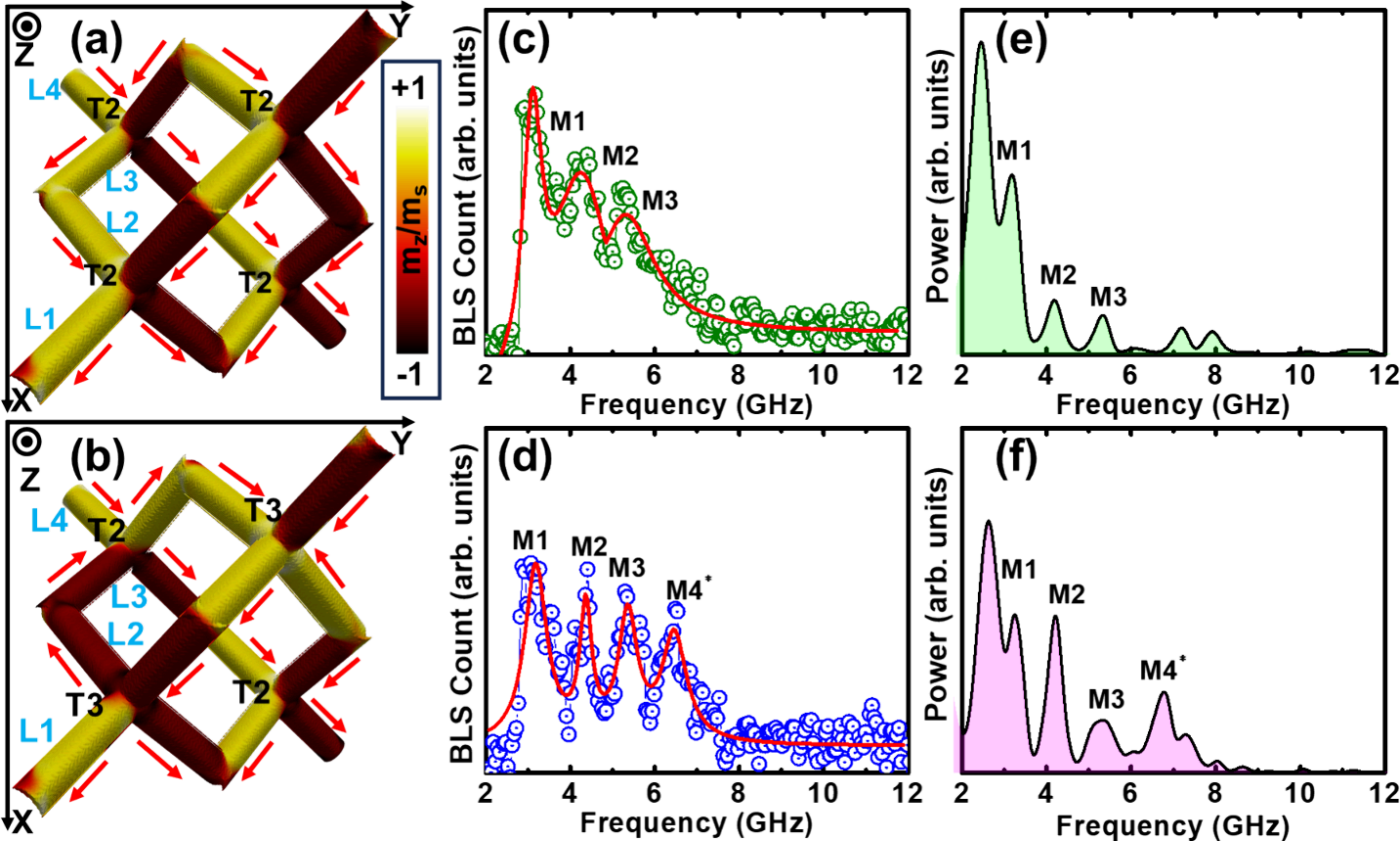
Ground-state magnetic microstate representing the unit cell of diamond bond lattice for the magnetized (a) and demagnetized (b) sample, where the red arrows indicate the spin direction. The color of the spins represents their orientation relative to the *z*-axis, as indicated by the color bar located on the right side of (a), where m_*z*_/m_s_ represents the normalized Z component of magnetization. The sublattices (L1, L2, L3 and L4) have been indicated in light blue color and the vertex type (T2 and T3) in black color. Experimental BLS spectra highlighting the presence of three SW modes in the magnetized sample **(**c) and four SW modes in the demagnetized sample (d). The simulated SW spectra from the simulated unit cell of magnetized sample (e) and demagnetized sample (f). Open circles in (c) and (d) represent the experimental data points, while the red and pink solid lines correspond to the results of the multipeak fitting.

To provide a general physical interpretation beyond the micromagnetic simulations, we consider the role of vertex symmetry and internal-field distribution. In a charge-neutral T2 vertex, the two-in/two-out configuration preserves a higher degree of symmetry in the local magnetostatic field, leading to more symmetric mode profiles and partial degeneracy of SW excitations across equivalent arms. In contrast, a T3 vertex contains a net magnetic charge, which introduces an imbalance in the local demagnetizing field and reduces the symmetry of the magnetic environment. This symmetry reduction modifies the internal-field landscape at and near the vertex, giving rise to a degeneracy lifting of modes and the emergence of additional localized or hybridized SW modes. Consequently, the appearance of an additional mode in the demagnetized state can be understood as a generic outcome of charge-induced symmetry lowering and field inhomogeneity, rather than a feature specific to a particular microstate.

The SW excitation characteristics in the magnetized and demagnetized samples differ in their inherent spatial excitation patterns as shown in the spatial power profiles in Figure 3. These were obtained by analyzing the dynamic magnetization components using the custom-built MATLAB-based code DOTMAG.^45^ Figure 3(a) presents the spatial power distribution of SW modes along the L1 sublattice, where the M2 and M3 modes exhibit symmetric power in the adjacent arms for the T2 vertex (dotted line) but show clear asymmetry between the two arms for the T3 vertex. This effect arises directly from the change in vertex type (T2 to T3) and reflects a vertex-dependent phenomenon. The spatial power profiles along the L2 sublattice (Figure 3(b)) indicate that most SW modes in both T2 and T3 vertices are localized with power concentrated near the vertex, while the M4 mode in the T3 vertex is more broadly distributed along the tetrapod arms. The cross-sectional power distributions near the vertex for the L2 sublattice (Figure 3(c)) in both T2 and T3 vertices reveal that low-frequency SW modes are concentrated at the edges and inner surfaces, while higher-frequency modes show enhanced power within the cross-section. This behavior reflects a general feature arising from the nontrivial crescent-shaped cross-section of the 3D-ASI. The corresponding phase profiles in the L1 and L2 sublattices, illustrating the distinct quantization characteristics of the T2 and T3 vertices, are discussed in Section V of the Supporting Information.

**Figure 3:**
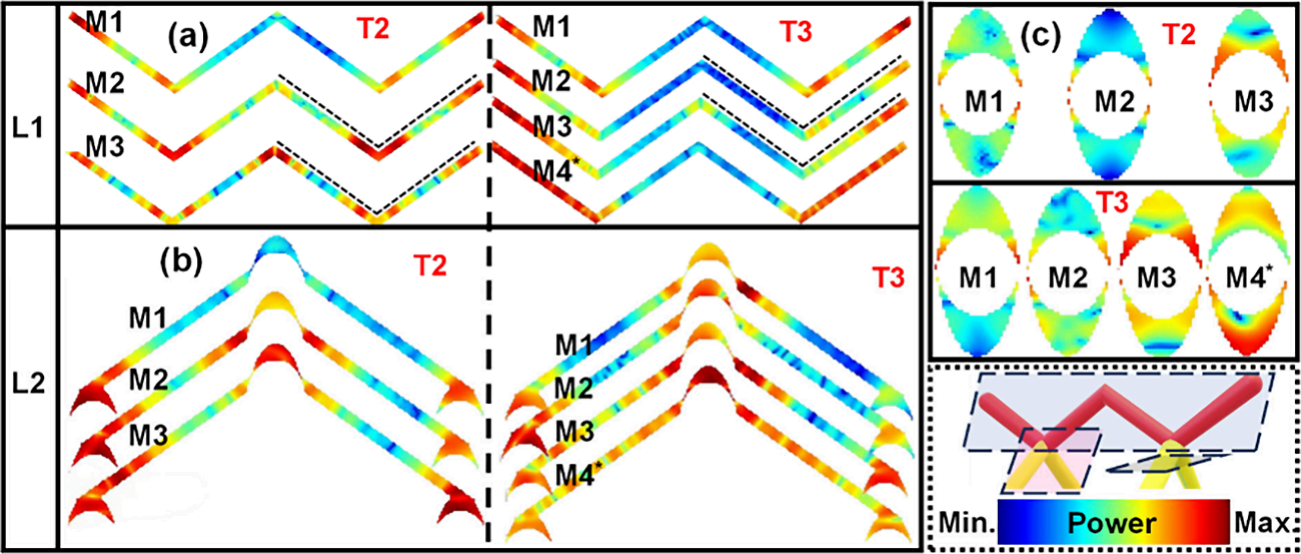
SW power profiles of the L1 (a) and L2 (b) sublattice along the length with T2 and T3 vertices. (c) SW power distributions of the L2 sublattice along cross-section near vertex, featuring T2 and T3 vertices. The schematic illustration of the cross sections, along with the corresponding color map for the power profile, is provided in the bottom-right corner.

In addition to charge-dependent SW excitation, exploring the reconfigurable magnonics through configurational anisotropy is particularly compelling, as even subtle changes in the direction of the *H* produce significant variations in internal field distribution, leading to complex and highly tunable SW dynamics in magnonic crystals.^46–48^ The direction-dependent tunability of physical properties is intrinsic to this 3D nanomagnetic structure due to its complex geometry.^40,49^ This behavior is illustrated in Figures 4(a) and 4(b), which show the simulated magnetic microstates at *H* = 1250 Oe for *H* applied along the L1 and L2 sublattices, respectively. For *H* applied along L1, the *H* has a component along the easy axes of the L1 and L3 sublattices, aligning their magnetic moments with the wire axis, while L2 and L4 experience the *H* along their hard axes. Owing to the nontrivial crescent-shaped cross-section, this leads to an unsaturated, spin-canted state resulting from the competition between shape anisotropy and the *H*. The configuration is reversed when *H* is applied along L2 sublattice (Figure 4(b)), as *H* is now along the hard axis for L1 and L3, and a component of *H* aligns with the easy axis of the L2 and L4 sublattices. The field evolution of the magnetic microstate for *H* applied along the L1 and L2 sublattices is shown in Supporting Information Figure S6. It indicates that the sublattices having easy axes along the *H* remain magnetized along the wire axis throughout the *H* variation. Whereas, the sublattices magnetized along the hard axis exhibit a gradual reduction in canting with decreasing *H* due to shape anisotropy and eventually become aligned along the wire direction, forming T2-type vertices. The demagnetizing-field distribution at *H* = 1250 Oe and the field-evolution of the demagnetizing energy density from 2000 to 0 Oe are shown in Supporting Information Figure S7. The results indicate that the demagnetizing-field is predominantly concentrated in the unsaturated sublattices, which are therefore termed spin-canted sublattices (SCS). In contrast, the demagnetizing-field is comparatively weak in the sublattices aligned with the *H*, referred to as spin-aligned sublattices. Moreover, the demagnetizing energy-density exhibits a direction-dependent decrease with decreasing *H*. This anisotropic evolution arises from the complex 3D geometry of the diamond-bond lattice.

**Figure 4:**
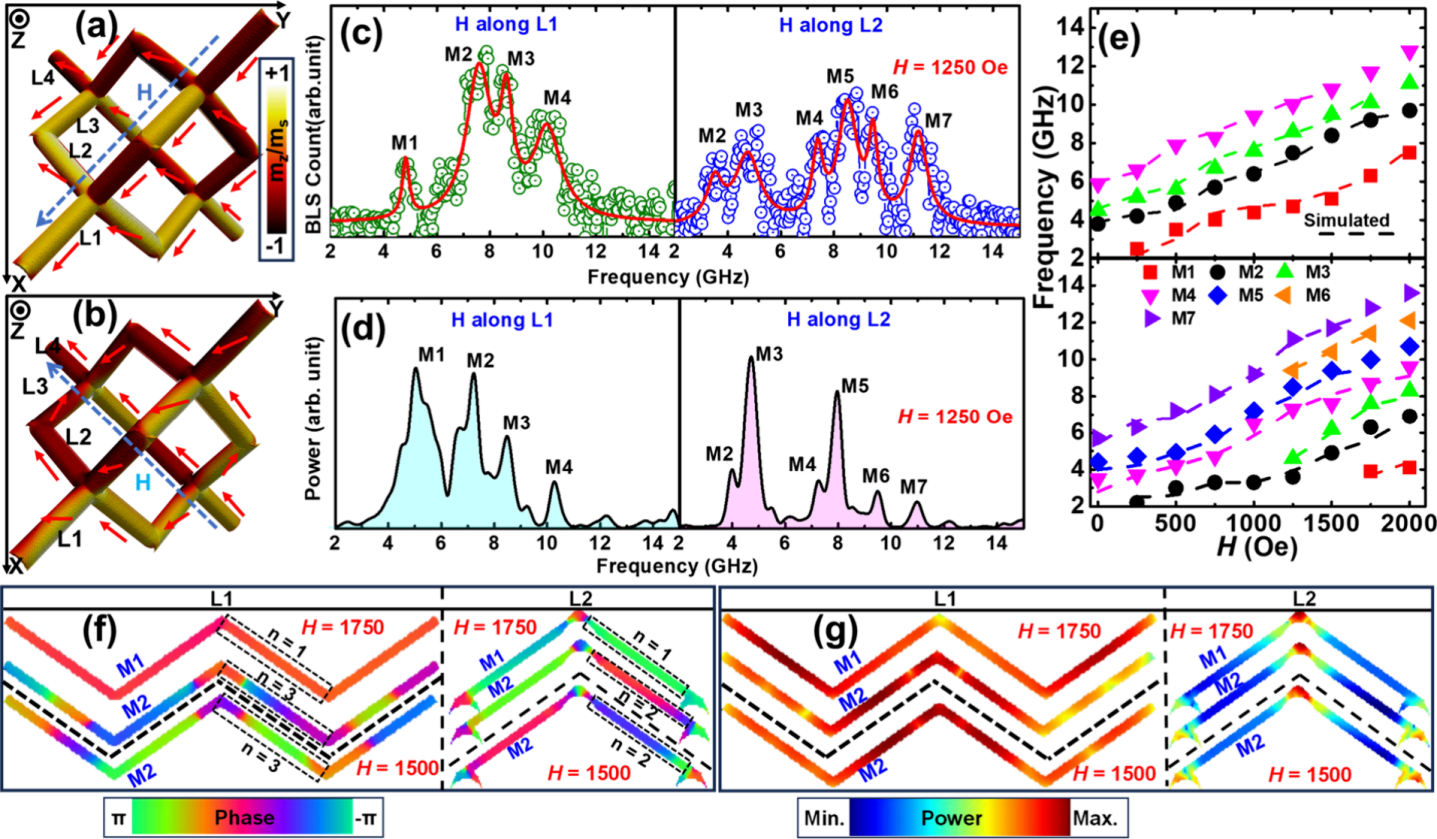
Ground-state magnetic microstate at *H* = 1250 Oe when *H* is applied along L1 (a) and L2 (b) sublattices, where the red arrows indicate the average spin direction. The corresponding color bar is provided on the right side of (a), where m_*z*_/m_s_ represents the normalized Z component of magnetization. The sublattices have been indicated in black color and the direction of *H* is in blue color. (c) The experimental BLS spectra revealing the presence of SW modes at *H* = 1250 Oe, when *H* is applied along L1 and L2 sublattices. Open circles represent the experimental data points, while the red and pink solid lines correspond to the results of the multipeak fitting. (d) Simulated SW spectra obtained at *H* = 1250 Oe when *H* is applied along L1 and L2 sublattices. (e) SW field dispersion measured for two different *H* directions, when *H* is applied along L1 (top panel) and L2 (bottom panel) sublattices. The simulated phase (f) and power (g) profiles of the M1 and M2 modes at *H* = 1750 Oe, and the M2 mode at *H* = 1500 Oe. The black dotted line in (f) and (g) separates the M1 and M2 modes at *H* = 1750 Oe from the M2 mode at *H* = 1500 Oe. The corresponding color maps for the power and phase profiles are provided at the bottom of the figure.

The configuration-dependent field-evolution of the magnetic microstates gives rise to configuration-dependent SW dynamical characteristics, as evident in the experimental BLS spectra at *H* = 1250 Oe (Figure 4(c)). Four SW modes are observed when the *H* is applied along L1, which increases to seven when the *H* is applied along L2. These features are qualitatively reproduced by the micromagnetic simulations, as shown in Figure 4(d). Minor discrepancies, such as the prediction of additional modes and differences in mode resolution, are unavoidable due to intrinsic differences between simulations and experiments for 3D systems, as discussed in the literature.^19,41^ The measured field-dispersion reveals a remarkable difference in SW characteristics on tuning the direction of *H* as shown in Figure 4(e). When the *H* is along the L1 sublattice, four consistent modes are observed throughout the variation of *H* from 2000 to 0 Oe. In contrast, when the *H*-direction is along L2 sublattice, additional features emerge (Figure 4(e)), including the disappearance of the lowest-frequency mode M1 below 1750 Oe and the merging of modes M2 with M3 and M5 with M6 at 1000 Oe. These features are confirmed by the experimental BLS-spectra at different *H* values (see Supporting Information Figure S8) and the corresponding simulated mode profiles. The phase profiles of the M1 and M2 modes at 1750 Oe and the M2 mode at 1500 Oe, taken at the cross-section of L1 and L2 sublattice, are shown in Figure 4(f). The quantization number n (dotted box) distinguishes the M1 and M2 modes at 1750 Oe and confirms the consistent nature of the M2 mode at both fields. The corresponding power map (Figure 4(g)) reveals stronger SW excitation in the L1 sublattice than in L2, indicating that SWs are predominantly excited in the SCS. This behavior can be attributed to the nontrivial crescent-shaped cross-section of the 3D-ASI. The generality of this trend is further verified by the power and phase analysis of each mode at *H* = 750 Oe for *H* along L1 and L2, as detailed in Supporting Information Section IX. The mode profiles confirming the merging of the M1-M2 and M5-M6 modes are also shown in Supporting Information Figure S9.

In summary, we demonstrate experimentally accessible and semiquantitative charge-sensitive spectral signatures in the SW dynamics of 3D-ASI, as probed by BLS spectroscopy and supported by micromagnetic simulations. Charged and charge-neutral vertices generate distinct SW spectra, establishing charge-dependent SW excitation as a robust, noninvasive probe of magnetic charge. Unlike static magnetic imaging, the SW spectrum provides a dynamic fingerprint of charge states, enabling readout in buried or otherwise inaccessible 3D nanomagnetic networks. Magnetic charge engineering enables zero-field tuning of SW characteristics, providing a route to field-free reconfigurable magnonic functionality. Field-dependent measurements further reveal pronounced configurational anisotropy, with the number, dispersion, and evolution of SW modes tuned by the orientation of the applied field relative to the lattice. Although demonstrated in a diamond-bond lattice, this charge- and configurational-anisotropy-controlled SW mechanism is expected to generalize to other 3D-ASI and nanomagnetic network architectures, opening pathways toward reprogrammable 3D magnonic and spintronic devices.

## Supplementary Material



## Data Availability

Information on the data underpinning this publication, including access details, can be found in the Cardiff University Research Data Repository at 10.17035/cardiff.32117719.

## Notes

Sample fabrication and characterization, measurement techniques, robustness of the BLS spectra, demagnetizing field and energy density profile (T2 and T3 vertices), spin-wave phase profile (T2 and T3 vertices), field evolution of magnetic microstate, field evolution of spin wave modes, spin-wave power and phase profile (*H* = 750 Oe) (PDF)
