# Supplementary material for: Magnetic Charge Fingerprints in the Spin-Wave Spectrum of Three-Dimensional Artificial Spin Ice
Source: Nano Lett. 2026 May 4;26(19):6363–9. doi: 10.1021/acs.nanolett.6c00670 (PMC13195663; doi:10.1021/acs.nanolett.6c00670)
Supplement: Supplementary file 1 [file nl6c00670_si_001.pdf]

Supporting Information

Magnetic Charge Fingerprints in the Spin-Wave Spectrum of Three-Dimensional Artificial Spin Ice

Chandan Kumar[1], Amrit Kumar Mondal[2], Sreya Pal[1], Sayan Mathur[1], Jay R. Scott[3], Arjen van Den Berg[4], Adekunle O. Adeyeye[4], Sam Ladak[4*], and Anjan Barman[1,5*]

[1]Department of Condensed Matter and Materials Physics, S. N. Bose National Centre for Basic Sciences, Block JD, Sector III, Salt Lake, Kolkata 700106, India

[2]Technical Research Centre, S. N. Bose National Centre for Basic Sciences, Block JD, Sector III, Salt Lake, Kolkata 700106, India

[3]Department of Physics, Durham University, Durham DH1 3LE, United Kingdom

[4]School of Physics and Astronomy, Cardiff University, Cardiff CF24 3AA, United Kingdom

[5]Department of Physics, School of Natural Sciences, Shiv Nadar Institution of Eminence (Delhi NCR), Dadri, UP 201314, India

*E-mail: abarman@bose.res.in; ladaks@cardiff.ac.uk

(I) Sample Fabrication and Characterization

The three-dimensional artificial spin ice (3D-ASI) lattices were produced using Two-Photon Lithography (TPL) involving a 780-nm femtosecond pulsed laser focused to a diffraction limited spot where a photoresist is polymerized. Using a set of galvo mirrors, the focal spot is translated through the resist to trace the desired geometry. The glass substrate was prepared with a 20-minute acetone bath in an ultrasonic cleaner followed by a 20-minute isopropanol bath in the ultrasonic cleaner and subsequently dried using compressed air. Immersion oil was drop-cast on one side of the coverslip along with a negative-tone photoresist (IPL, proprietary to NanoScribe GMBH) drop-cast on the reverse side. The coverslip is loaded into the TPL system, and a script outlining the desired geometries is executed. After exposure, the samples were placed in a propyl glycol monomethyl ether acetate (PGMEA) developer bath to remove unexposed resist, followed by an isopropyl alcohol (IPA) bath and then gently dried using compressed air.

Four 15-nm gold layers were deposited using thermal evaporation at a 20° angle, with the sample stage rotated 90° between each evaporation. Finally, 0.067 g of permalloy ($Ni_{81}Fe_{19}$) was deposited to yield a 44-nm permalloy layer. The base pressure for all evaporations was below 10^{-6} mbar. SEM was carried out using a Hitachi Regulus 8230 SEM.

Magnetic Force Microscopy (MFM) measurements were performed using the Bruker Dimension ICON scanning probe microscope in tapping mode using supersharp ultra-low moment probes from NANOSENSORS magnetized using a 0.5 T permanent magnet. The samples were positioned such that the L1 sublattice is parallel to the cantilever, and the scan direction set to 45°. The lift mode scans for obtaining magnetic contrast were performed at a 130-nm lift hight, allowing for higher drive amplitudes for improved magnetic contrast.

Magnetization and Demagnetization: The magnetized sample was obtained by applying an in-plane magnetic field, of magnitude 100 mT along the projection of L1. For demagnetized sample, we used a protocol typically used in 2D-ASI, based on method 1 outlined in prior work[1]. An in-plane oscillating field is applied to the sample, starting at 0 mT and ramping up to 75 mT at 2.5 T/s, the amplitude of the oscillation decays to 0 mT over five days. Effective field rotation is achieved by rotating the sample at 1000 rpm about the axis perpendicular to the field. The atomic force microscopy (AFM) and MFM images captured at the remanent state for both magnetized and demagnetized samples are shown in Figure S1.

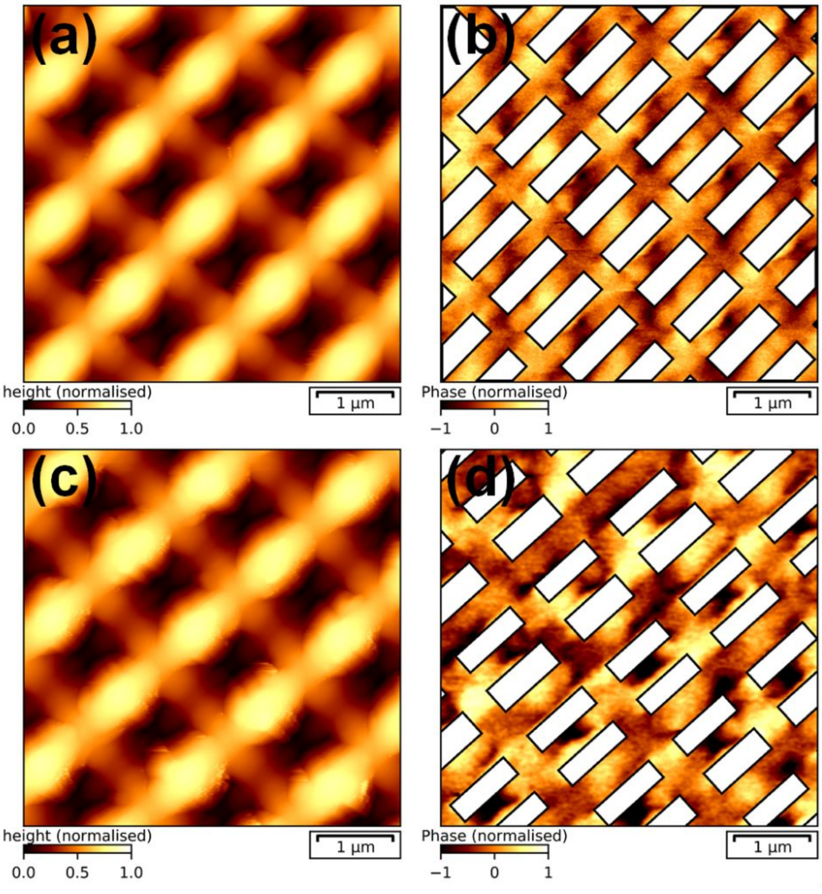

Figure S1. AFM and corresponding MFM images acquired at the remanent state for the magnetized sample (a, b) and the demagnetized sample (c, d).

(II) Measurement Techniques

Brillouin Light Scattering (BLS) Spectroscopy: The spin-wave (SW) dynamics of the 3D-ASI samples were measured using conventional BLS, a non-contact and non-invasive technique ideal for detecting thermally excited SWs at room temperature without external excitation. BLS operates on the principle of inelastic light scattering, described quantum mechanically as a photon-quasiparticle (magnon here) interaction, where the creation (Stokes process) and annihilation (anti-Stokes process) of magnons are observed. The BLS spectra were obtained in the Damon–Eshbach (DE) geometry using a Sandercock-type (3+3)-pass tandem Fabry-Pérot interferometer and a p-polarized single-longitudinal-mode solid-state laser (532 nm wavelength, 230 mW power). The laser power on the sample surface was 65 mW, with a spot size of ~50 μm in diameter, closely matching the sample's lateral dimensions [2]. This setup enabled SW measurements from nearly the entire sample volume. Cross-polarization between the inelastically backscattered and incident beams was used to minimize phonon interference. The sample was mounted on a 360° in-plane rotating stage, allowing precise rotation along the desired direction within the plane of the substrate. A permanent magnet was used to apply the *H*, and its strength was accurately measured at each step of the measurement to obtain the field-dispersion. BLS measurement geometry, illustrating the technique, sample structure, and representative BLS spectra, is presented in Figure S2.

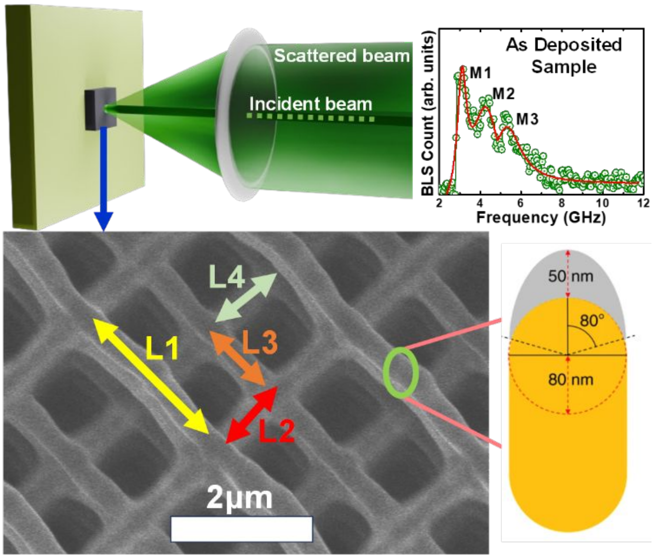

Figure S2. Schematic representation of the BLS measurement geometry, along with an SEM image of the sample, highlighting the different sublattices indicated by coloured arrows and includes sample details.

Micromagnetic simulation: The micromagnetic simulations were performed using the GPU-accelerated micromagnetic simulation package mumax3[3]. We utilized cuboidal cells with dimensions of 5×5×5 nm³, which is less than the exchange length of permalloy (≈ 5.2 nm). The material parameters used in the simulation were: gyromagnetic ratio (γ) = 17.6 MHz/Oe, saturation magnetization (M_s) = 860 emu/cc, anisotropy field (H_K) = 0, and exchange stiffness constant (A_{ex}) = 13×10^{-7} erg/cm for permalloy[4]. To simulate the experimental charge configuration, we designed a unit cell of the diamond bond lattice composed of crescent-shaped nanowires with dimensions similar to the experimental sample and applied a 2D periodic

boundary condition in the x−y plane. The simulated structure along the z-direction contains four sublattices, similar to the experimental sample. The magnetization of each wire was defined separately to mimic the charge configurations obtained experimentally for the magnetized and demagnetized samples and were relaxed to obtain the equilibrium magnetic microstate. For the simulation of SW dynamics, we applied a square-shaped pulsed magnetic field with a peak amplitude of 5 Oe along the z-direction, a rise and fall time of 10 ps each, and a duration of 20 ps to the equilibrium magnetic state using a Gilbert damping parameter (α) = 0.008[2]. The SW spectra were calculated by performing a fast Fourier transformation (FFT) of the x-component of the dynamic magnetization (m_x). To mimic the field-dispersion results, the external field was applied parallel and perpendicular to the L1 sublattice and allowed to relax to obtain the equilibrium magnetic microstates. Thereafter, the SWs were excited using the same technique mentioned above, except that the amplitude of the square-shaped pulsed magnetic field was increased to 20 Oe.

(III) Robustness of the BLS Spectra

The robustness of the additional mode in the demagnetized state has been verified using multiple fitting approaches, including single- and multi-peak Gaussian and Lorentzian functions. In all cases, the mode is consistently well resolved, as shown in Figure S3. The extracted peak frequencies and peak-width (full width at half maximum) values, summarized in Table S1, further confirm that the peaks are well separated and lie outside each other's uncertainty ranges.

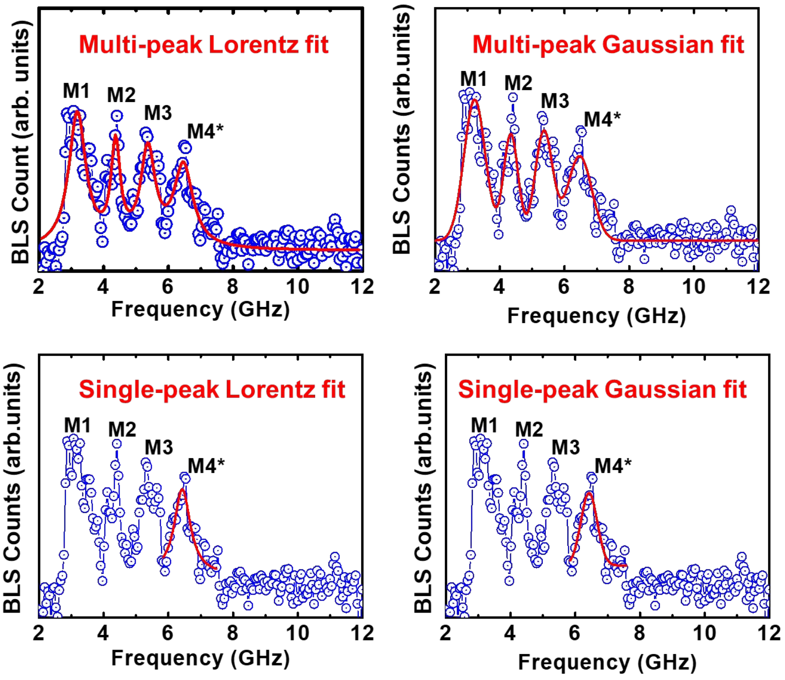

Figure S3. Single- and multi-peak Gaussian and Lorentzian fits of the additional spin-wave mode observed in the demagnetized sample.

Table S1. Peak frequencies and peak width of each mode in the demagnetized sample obtained from multi-peak Gaussian and Lorentzian fitting.

		M1	M2	M3	M4
**Multi-peak Gaussian fit**	**Peak Frequency (GHz)**	3.20 ± 0.02	4.35 ± 0.02	5.35 ± 0.03	6.46 ± 0.04
	**Peak Width (GHz)**	0.66 ± 0.04	0.45 ± 0.05	0.55 ± 0.06	0.70 ± 0.05
**Multi-peak Lorentzian fit**	**Peak Frequency (GHz)**	3.18 ± 0.02	4.37 ± 0.02	5.37 ± 0.03	6.46 ± 0.03
	**Peak Width (GHz)**	0.62 ± 0.06	0.34 ± 0.06	0.54 ± 0.07	0.68 ± 0.02

(IV) Demagnetizing Field and Energy Density Profile of T2 and T3 Vertices

The demagnetizing field distributions of the T2 and T3 vertices are presented in Figure S4, revealing a clear contrast between the two configurations. It is evident that the demagnetizing field is significantly more concentrated at the T3 vertex compared to T2. This difference is further reflected in their energy characteristics, as summarized in Table S2. Specifically, both the total and demagnetizing energy densities of the T3 vertex are approximately 20% higher than those of T2.

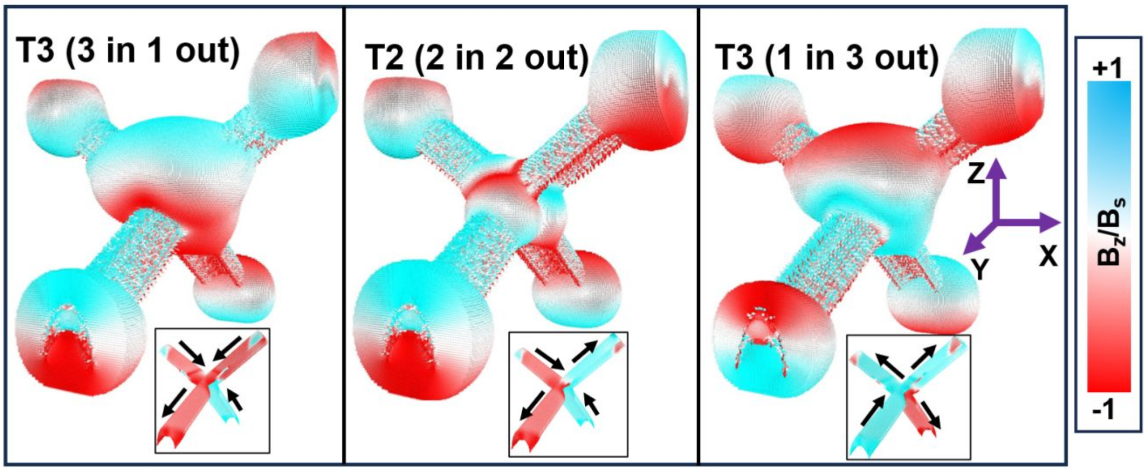

Figure S4. Demagnetizing field distributions of T3 and T2 vertices. The corresponding colour map is provided on the right side of the figure, where Bz/Bs represents the normalized Z component of demagnetizing field.

Table S2. Total and demagnetizing energy densities of the T2 and T3 vertices.

	T3 (3 in 1 out)	T2 (2 in 2 out)	T3 (1 in 3 out)
**Energy density (Total) (J/m^3)**	284.28	233.19	284.28
**Energy density (Demagnetizing) (J/m^3)**	262.78	218.19	262.78

(V) Spin-Wave Phase Profile of T2 and T3 Vertices

The phase profiles of SWs in the type-II (T2) and type-III (T3) vertices are illustrated below using a home-built MATLAB package DOTMAG[5]. The quantization numbers (n and n') of the SWs within the dotted box are shown for each mode. Notably, an asymmetry in the quantization of SW modes is observed in the T3 vertex within the L1 sublattice, both along the length (Figure S5(a)). In contrast, the T2 vertex exhibits symmetric quantization in the two adjacent arms of the same sublattice (L1). For the L2 sublattice, the SW quantization is symmetric along both the length (Figure S5(b)) and cross-section (Figure S5(c)) for both the T3 and T2 vertices. A consistent trend is that the 'n' increases with frequency, and the value of 'n' remains identical for the same SW modes in the T2 and T3 vertices within the L2 sublattice.

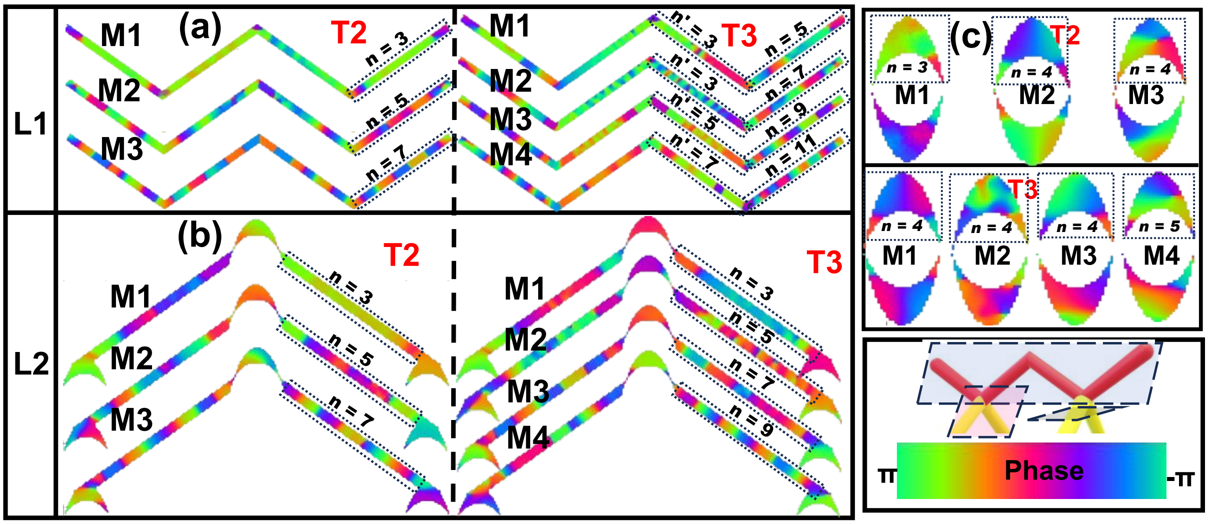

Figure S5. SW phase profiles of the L1 (a) and L2 (b) sublattice along the length with T2 and T3 vertices. SW phase distributions of the L2 sublattice along cross-section near vertex, featuring T2 and T3 vertices. The quantization number (n & n') within the dotted box has been indicated for each SW mode. The schematic illustration of the cross-sections, along with the corresponding color map for the phase profile, is provided in the bottom-right corner.

(VI) Field Evolution of Magnetic Microstate

The field evolution of magnetic microstates under application of *H* along the L1 and L2 sublattices is shown in Figures S6(a) and (b), respectively. Sublattices aligned with the *H* become magnetized along their length, while those perpendicular to the *H* exhibit canted spin configurations due to the competition between shape anisotropy and the *H*. At lower fields, shape anisotropy dominates, whereas increasing *H* strength progressively increases the canting in the sublattices perpendicular to *H*, as evident in Figures S6(a) and S6(b).

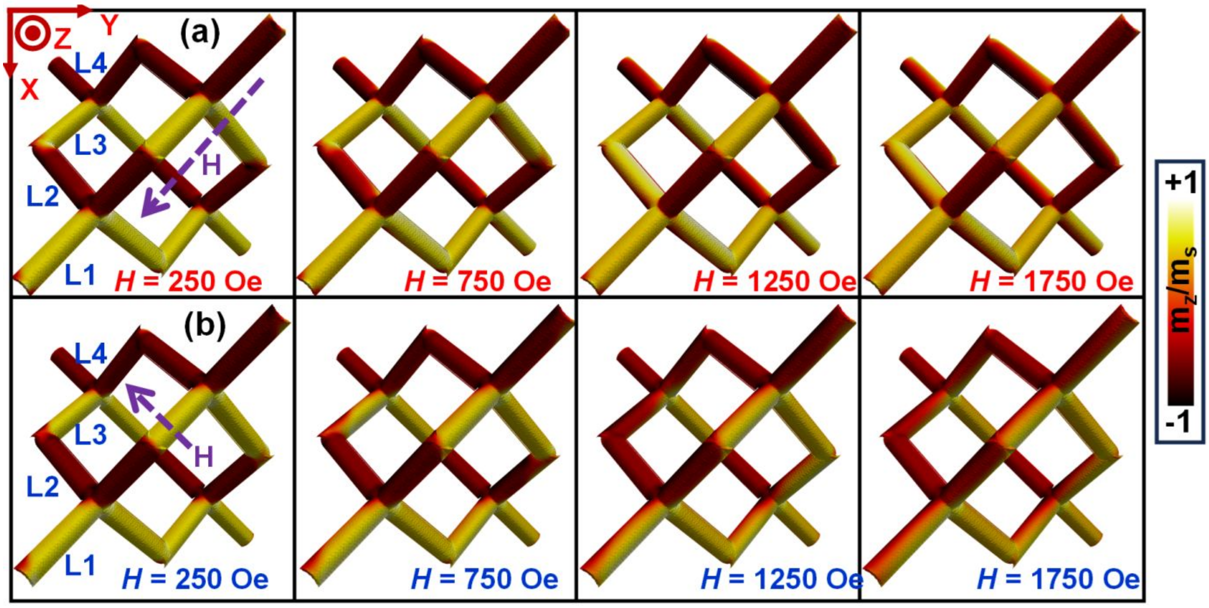

Figure. S6. The simulated ground-state magnetic microstate at $H = 250$, 750, 1250 and 1750 Oe, when *H* is applied along L1 (a) and L2 (b). The corresponding colour bar is provided on the right side of the figure, where m_z/m_s represents the normalized Z component of magnetization.

(VII) Demagnetizing Field Analysis

The demagnetizing field (B) exhibits both spatial and field-dependent variations depending on the direction of the applied field *H*. The spatial distributions of B for the cases where *H* is applied along the L1 and L2 sublattices are shown in Figures S7(a) and S7(b), respectively. These distributions highlight significant differences in B arising from the direction of *H*. The field evolution of the demagnetizing energy density for *H* applied along L1 and L2 is presented in Figure S7(c), clearly demonstrating the strong direction dependence in the field evolution of the demagnetizing energy density.

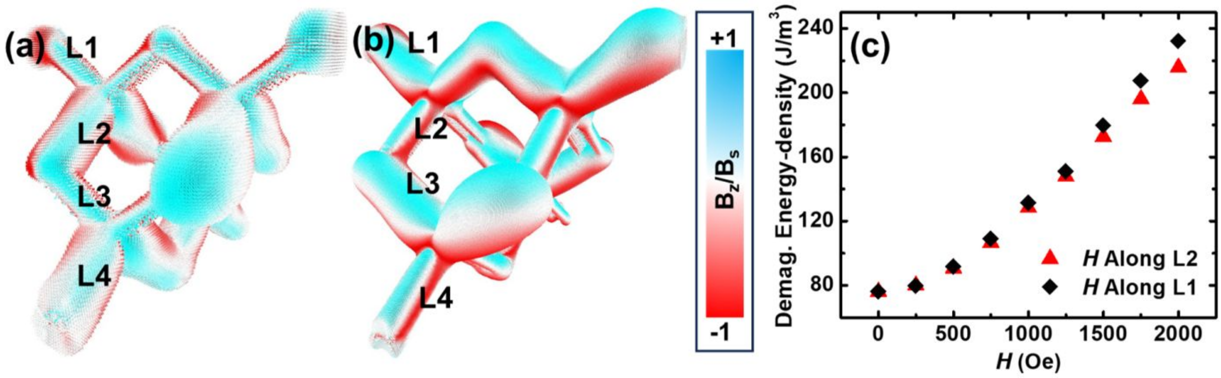

Figure. S7. The demagnetizing field distribution at H = 1250 Oe for H applied along the L1 (a) and L2 (b) sublattices. The corresponding colour map is provided on the right side of the figure S7(b), where Bz/Bs represents the normalized Z component of demagnetizing field. A comparison of the field evolution of the demagnetizing energy density for H applied along the L1 and L2 sublattices.

(VIII) Field Evolution of Spin Wave Modes

The experimental BLS spectra for fields from H = 1750 to 1000 Oe, with H applied along the L1 and L2 sublattices, are presented in Figures S8(a) and S8(b), respectively. The obtained spectra confirm the presence of four consistent modes at different fields when H is applied along L1. In contrast, for H applied along L2, the spectra show the termination of the M1 mode at H = 1500 Oe and the merging of the M2-M3 and M5-M6 modes around 1000 Oe.

The mode profiles of the M5 and M6 modes at H = 1250 Oe and of the M5′ mode at H = 1000 Oe are shown in Figure S9(a). These results indicate that the M5′ mode, which emerges from the merging of the M5 and M6 modes at H = 1000 Oe, retains the characteristic features of the original M5 mode. A similar analysis for the merging of the M2 and M3 modes at H = 1000 Oe is presented in Figure S9(b), which highlights the evolution of the new M2′ mode at H = 1000 Oe with a more extended nature compared to M2 and M3.

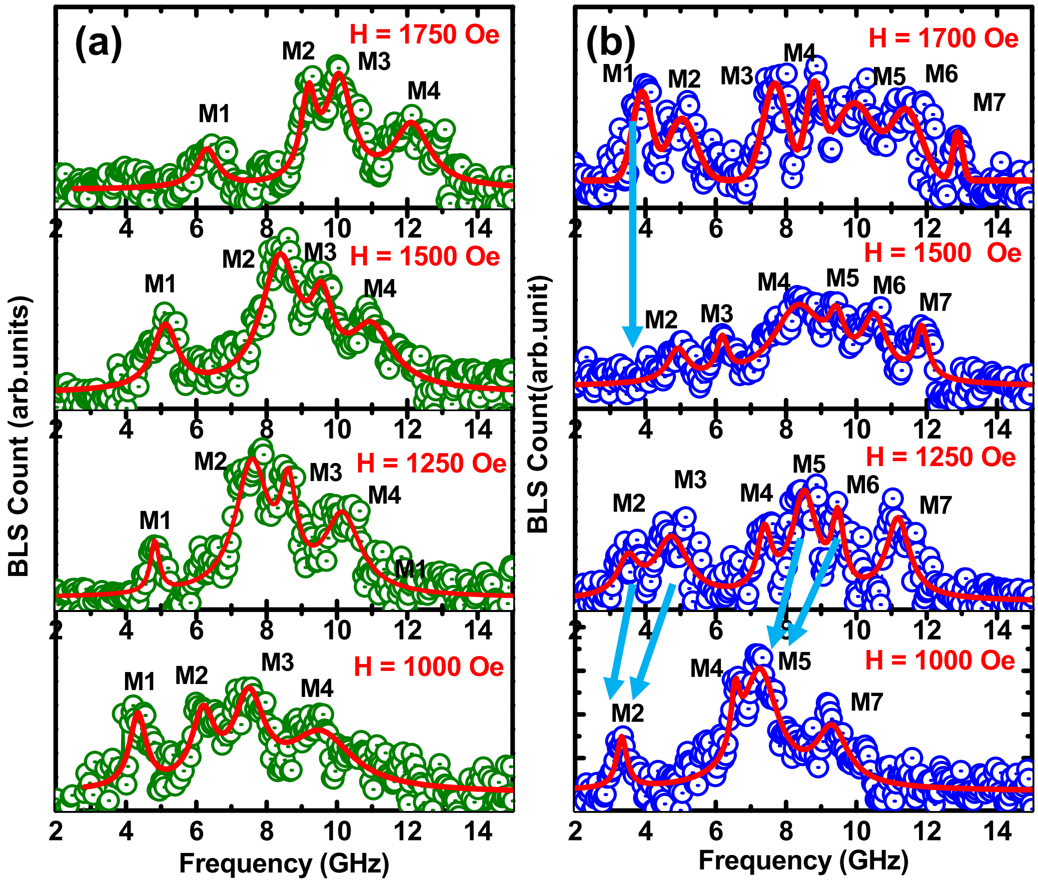

Figure. S8. The experimental BLS spectra at different *H* values, revealing the field evolution of the SW modes, when *H* is applied along L1 (a) and L2 (b) sublattices. Open circles represent the experimental data points, while the red and pink solid lines correspond to the results of the multipeak fitting.

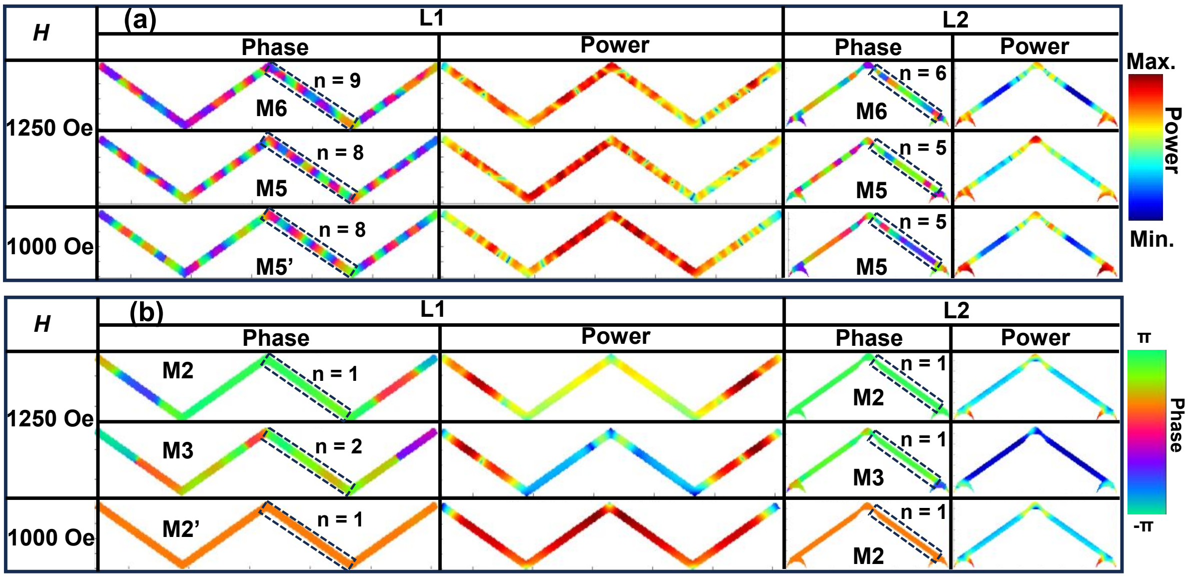

Figure. S9. The power and phase profile along L1 and L2 sublattices highlighting the mode merging of M5-M6 (a) and M2-M3 (b) at H = 1000 Oe. The corresponding colour map is displayed on the right side of the figure.

(IX) Spin-Wave Power and Phase Profile at H = 750 Oe

The power profiles of the SW modes at H = 750 Oe for fields applied along the L1 and L2 sublattices are presented in Figure S10, revealing a higher concentration of SW power in the spin-canted sublattices (L2 for H applied along L1 and L1 for H applied along L2) compared to the spin-aligned sublattices (L1 for H applied along L1 and L2 for H applied along L2). The phase profiles of the SW modes at H = 750 Oe along the L1 and L2 sublattices are presented in Figure S11, revealing quantization of the SWs. Notably, the quantization, denoted by 'n', is higher in the spin-canted sublattices than in the spin-aligned sublattices.

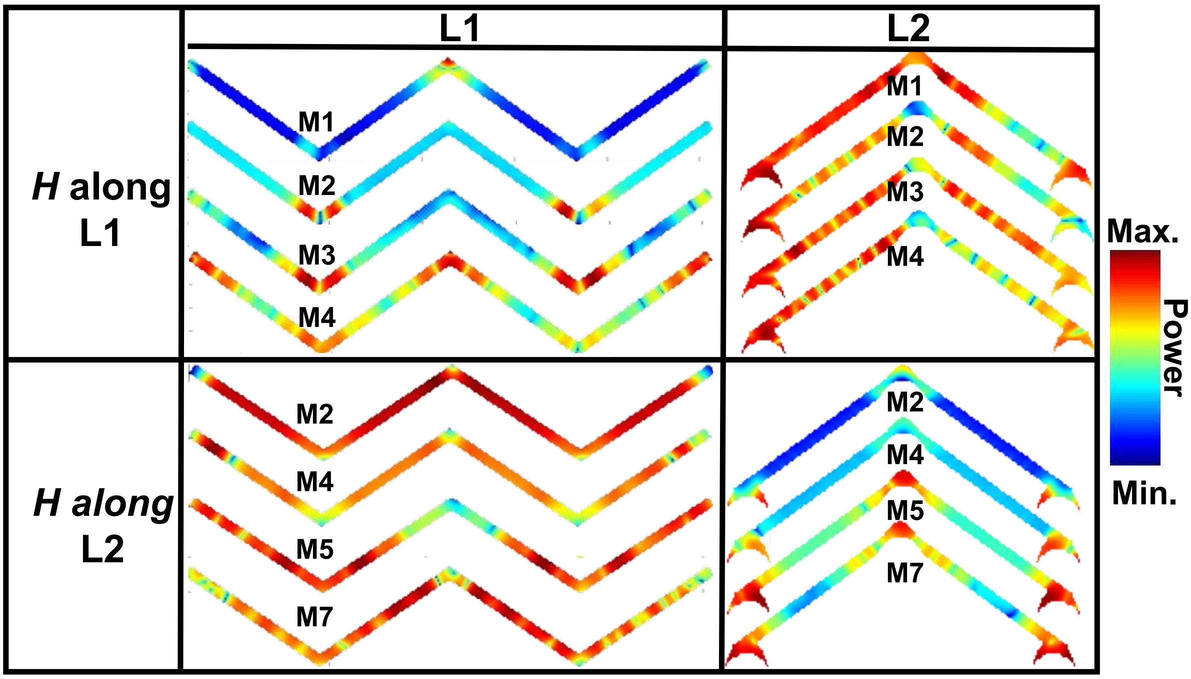

Figure. S10. SW power profile (at H = 750 Oe) along the L1 and L2 sublattices. The corresponding colour map is displayed on the right side of the figure.

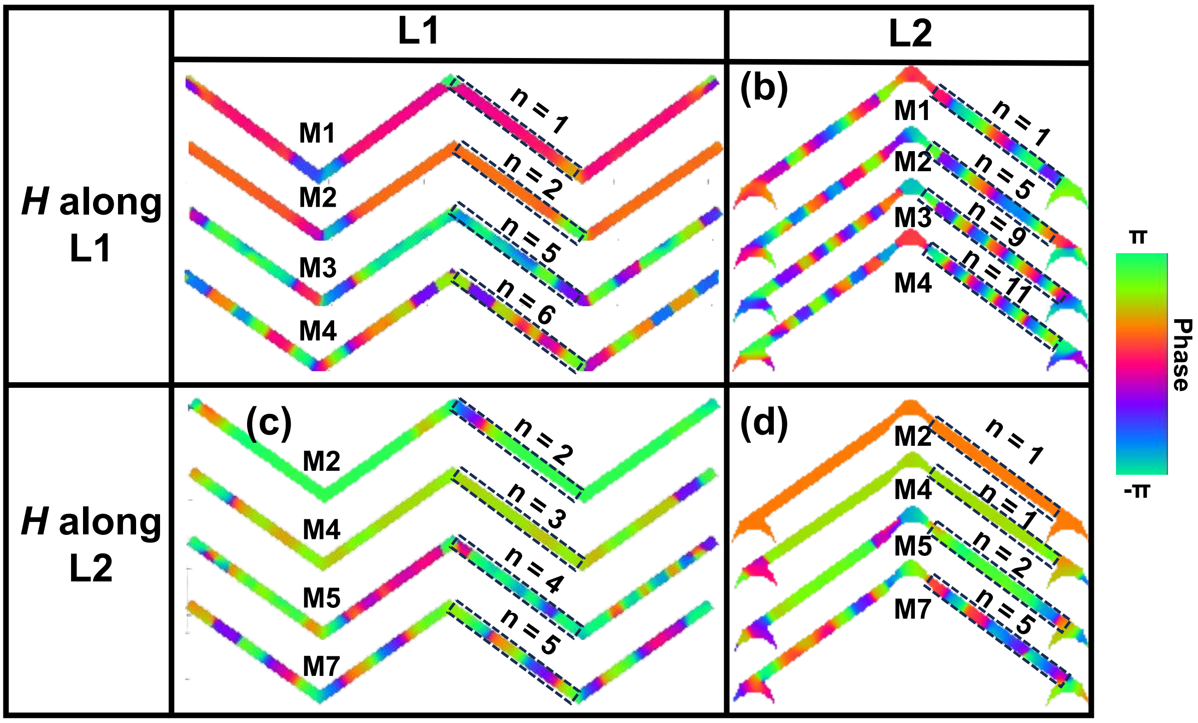

Figure. S11. SW phase profile (at H = 750 Oe) along the L1 and L2 sublattices. The corresponding colour map is displayed on the right side of the figure.

Reference

(1) Wang, R. F.; Li, J.; McConville, W.; Nisoli, C.; Ke, X.; Freeland, J. W.; Rose, V.; Grimsditch, M.; et al. Demagnetization protocols for frustrated interacting nanomagnet arrays. *J. Appl. Phys.* **2007**, *101* (9).

(2) Sahoo, S.; May, A.; van Den Berg, A.; Mondal, A. K.; Ladak, S.; Barman, A. Observation of Coherent Spin Waves in a Three-Dimensional Artificial Spin Ice Structure. *Nano Lett.* **2021**, *21* (11), 4629-4635.

(3) Vansteenkiste, A.; Leliaert, J.; Dvornik, M.; Helsen, M.; Garcia-Sanchez, F.; Van Waeyenberge, B. The design and verification of MuMax3. *AIP Advances* **2014**, *4* (10), 107133.

(4) Kumar, C.; Pal, P. K.; Barman, A. Spin-wave mode reversal and anisotropy in bicomponent magnonic crystals. *Phys. Rev. B* **2024**, *109* (7), 075407.

(5) Kumar, D.; Dmytriiev, O.; Ponraj, S.; Barman, A. Numerical calculation of spin wave dispersions in magnetic nanostructures. *J. Phys. D: Appl. Phys.* **2012**, *45* (1), 015001.
